# Simultaneous Determination of 13 Organic Acids in Liquid Culture Media of Edible Fungi Using High-Performance Liquid Chromatography

**DOI:** 10.1155/2020/2817979

**Published:** 2020-07-31

**Authors:** Changxia Yu, Yan Wang, Hui Cao, Yan Zhao, Zhengpeng Li, Hong Wang, Mingjie Chen, Qingjiu Tang

**Affiliations:** Institute of Edible Fungi, Shanghai Academy of Agricultural Sciences, Key Laboratory of Edible Fungi Resources and Utilization (South), Ministry of Agriculture, PR China, National Engineering Research Center of Edible Fungi, Shanghai 201403, China

## Abstract

The study aimed at detecting 13 organic acids (oxalic acid, maleic acid, citric acid, tartaric acid, malic acid, quinic acid, succinic acid, fumaric acid, formic acid, acetic acid, propionic acid, isobutyric acid, and butyric acid) by establishing a high-performance liquid chromatography (HPLC). The analysis was performed using two sugar columns, i.e., SH1011 column and KC-811 column. The optimal conditions were as follows: 4 mmol/L HClO_4_ solution as the eluent with UV-visible detector (210 nm), a flow rate of 1 mL/min at the temperature of 60°C, and the injection volume at 10 *μ*L. The results showed that all the calibration curves had excellent linearity (*R*^2^ > 0.9991) within the test ranges. The RSD values of the thirteen analytes were lower than 2.94% at three levels, the recoveries were 91.9%-102.0%, the limit of detection (LOD) was between 0.05 and 10.63 *μ*g/mL, and the quantification (LOQ) was between 0.10 and 19.53 *μ*g/mL. Finally, the proposed methodology was successfully applied for the analysis of organic acids in the culture medium of edible fungi. In conclusion, the study findings proved that the method was sensitive, accurate, reproducible, and could be readily applied to analyze the organic acids in the samples.

## 1. Introduction

Several fungi can efficiently produce large quantities of metabolites during their growth process. The growth of edible fungi is characterized by the degradation of lignocellulose in the substrates. Degradation of these macromolecules of the raw materials releases metabolites such as water, carbon dioxide, and other low molecules (including organic acids) that are utilized as the nutritional sources by the fungi. However, the gradual accumulation of metabolites might affect the growth process (such as continuous decrease of pH in the substrates) of the edible fungi. Therefore, significant research on the metabolites of edible fungi is warranted. Low molecular weight organic acids are the primary metabolic products of the edible fungi and exhibit several essential metabolic features [1, 2]. However, very few reports on the mechanisms of these metabolic products in promoting edible fungal growth are available except oxalic acid. Oxalic acid is the primary extracellular secretion of fungi and widely acknowledged for its unique features, such as chelating metals [3, 4], reducing the pH value of host tissue by generating H^+^ through ionization [5–8]. Oxalic acid acts as an electron donor during the lignocellulose degradation and buffering the environment outside of fungal hyphae [9]. Accumulating studies have reported the effects of the organic acids on mycelia growth. Liu et al. [10] demonstrated that the addition of a certain amount of citric acid or succinic acid to the culture medium of *Agaricus bisporus* could promote the growth of mycelia. Similarly, Wang et al. [11] elucidated that the addition of an appropriate amount of oxalic acid, citric acid, or tartaric acid to a liquid culture medium could significantly increase the mycelial biomass of *Pleurotus eryngii* and *Flammulina velutipes*. To date, it is unclear whether organic acids are their valuable metabolites in the growth of edible fungi. Supporting the findings above, a method for the determination of the organic acids in the culture medium of the edible fungi has been established with great significance to study the dynamic changes and metabolic regulation of the organic acids during mycelia cultivation.

To the best of our knowledge, there are several analytical methods for the determination of organic acids, such as capillary electrophoresis, gas chromatography, ion chromatography, and reversed-phase high-performance liquid chromatography [12, 13]. However, each method has its disadvantages. For capillary electrophoresis, the reproducibility is low, and the results are not precise enough [14–16]. Gas chromatography requiring samples must be analyzed after derivatization [17–19]. Ion chromatography needs a long analyzing cycle and is not suitable for the rapid detection of samples [20–22].

High-performance liquid chromatography is the most reliable method due to its high sensitivity, excellent repeatability, simple operation, and can detect a variety of organic acids simultaneously. It has been widely used for the determination of organic acids in various foods and natural products [23, 24]. At present, most of the researches are revealing the effect of organic acids on their flavor through high-performance liquid chromatography. He et al. established a simultaneous HPLC method for the determination of tartaric acid, malic acid, acetic acid, and citric acid in *Lentinula edodes* [25]. Yang et al. separated and determined 7 kinds of organic acids in the fruiting bodies of 8 types of edible fungi through reversed-phase high-performance liquid chromatography and revealed the flavor characteristics of edible mushrooms [26]. However, researches on the detection of organic acids in the culture medium of edible fungi have rarely been reported so far. Therefore, we cross-checked the previous methodologies that used high-performance liquid chromatography for detecting the organic acids and found that most of them used amino columns. These amino columns have a reduced resolution for organic acid and can only detect a few types of organic acids that could not conform to the requirements of a comprehensive analysis [27–29].

Considering the above observations, we used the sugar columns that have a good resolution for detecting more organic acids and simultaneously chose 13 organic acids as the standard compounds to analyze the metabolic products. Two sugar columns, SH1011 column and KC-811 column, were adopted in series to set up a rapid and convenient method in this study. The contents of organic acids in the liquid medium of the edible fungi were also analyzed.

## 2. Materials and Methods

### 2.1. Instruments, Reagents, and Materials

All the analyses were performed on a Waters 2695 liquid chromatography system (Waters, Milford, USA) equipped with a vacuum degasser, a quaternary solvent deliver system, an autosampler, a column compartment, and a w2489 UV visible detector. SH-G guard column (50 mm × 6.0 mm, 7 *μ*m), SH1011 sugar column (300 mm × 8.0 mm, 6 *μ*m), and KC-811 sugar column (300 mm × 8.0 mm, 6 *μ*m) were purchased from Shodex (Tokyo, Japan), the stationary phases of the analytical column and the protective column were a high-capacity cation exchange resin formed by hydrogen-type sulfonated cross-linked polystyrene divinylbenzene copolymer. Deionized water was prepared using a Milli-Q ultrapure water purifier (ELGA, Labwater, Marlow, UK).

All the reagents used in this study were of analytical grade. Perchloric acid used for HPLC was purchased from Sinopharm Chemical Reagent Co., Ltd. (Shanghai, China). Thirteen types of standard substances, such as oxalic acid, maleic acid, citric acid, tartaric acid, malic acid, quinic acid, succinic acid, fumaric acid, formic acid, acetic acid, propionic acid, isobutyric acid, and butyric acid, were supplied by Sigma (St. Louis, MO, USA).

*L. edodes* strain 215 and *F. velutipes* strain G1 were provided by the Culture Collection Center of the Institute of Edible Fungi at the Shanghai Academy of Agricultural Sciences. The commercially available potato dextrose broth (PDB) (BD Company, USA), 24 g/L was used for the liquid medium, whereas the commercially available potato dextrose agar (PDA) (BD Company, USA), 39 g/L was used for the solid medium.

### 2.2. Optimization of Chromatographic Conditions

An SH1011 sugar column coupled with a KC-811 sugar column was used as analytical columns, and an SH-G column was used as a guard column. HClO_4_ solution was used as a mobile phase, and the substances were detected with a UV-visible detector. The chromatographic conditions, including absorption wavelength, the concentration of mobile phase, column temperature, and flow rate, were optimized as follows: to obtain a good resolution of the chromatograms and appropriate retention time of these organic acids, 13 standard solutions were analyzed to investigate the separation resolution through different wavelengths of 200 nm, 210 nm, 220 nm, and 430 nm. The concentration of the mobile phase was formulated for 1, 4, or 6 mmol/L, the column temperature was set at 40, 50, 60, and 70°C, and the flow rate was set at 0.5, 1.0, and 1.2 mL/min.

### 2.3. Experiment of Standard Curve

Each standard substance (maleic acid and fumaric acid were of 5 mg each, oxalic acid, citric acid, tartaric acid, malic acid, quinic acid, succinic acid, formic acid, acetic acid, propionic acid, isobutyric acid, and butyric acid were of 20 mg each) was dissolved in the deionized water in a 10 mL measuring flask, and then, diluted to a series of standard solutions of 2000, 1000, 500, 200, 100, 50, 20, 10, 4, 2, and 0.4 *μ*g/mL to obtain the calibration curves. Later, the relative correction factors were calculated.

### 2.4. Preparation of Sample Solutions

*L. edodes* strain 215 was subcultured on a PDA plate (25°C for 7 d). Then, six 9 mm diameter discs of mycelia culture were transferred to a blender cup containing 100 mL PDB and homogenized for 30 s. Aliquots (1 mL) were transferred to 250 mL flasks containing 100 mL PDB medium, and the flasks were incubated at 25°C, 150 r/min. Mycelia were removed through analytical filter paper, and the residual medium was centrifuged at 4°C, and 13400 g for 10 min, and the supernatant was filtered through a membrane filter (0.22 *μ*m). The sample solution of *F. velutipes* strain G1 was prepared according to the methods used for the preparation of *L. edodes* strain.

### 2.5. Determination of Samples

The cultured liquid mediums were used for the present study. Samples were prepared as described in 2.4. The contents of organic acids were calculated using the regression equation from the standard curve.

## 3. Results and Discussion

### 3.1. Optimization of HPLC Conditions

The HPLC conditions were optimized using standard solutions and samples. A total of 13 components were investigated by comparing the wavelengths of UV absorption. The results indicated that each organic acid had a maximum UV length of 210 nm, and the sharpness and the symmetrical resolution of the peak were better than the other wavelengths. Therefore, 210 nm was considered as the optimal detection wavelength. It is known that increasing the concentration of the mobile phase could increase the resolution, but in our case, the pH of 6 mmol/L HClO_4_ was only 2.11, which could destroy the columns severely. Similarly, the pH of 4 mmol/L HClO_4_ was 2.50 and exhibited a good separation for organic acids. Therefore, 4 mmol/L concentration of the mobile phase was chosen as the optimal concentration. By comparing the resolutions and peak shapes of the investigated components, 60°C was chosen as the optimal column temperature. Increasing the elution flow rate could run the retention time of quantitative compounds in advance, but the high flow rate would decrease the resolution, the low flow rate would prolong the analyzing time. After comparing the chromatograms, 1 mL/min of the elution flow rate was selected as the optimal flow rate. Therefore, the method established in this research using SH1011 coupled with KC-811, and 4 mmol/L HClO_4_ solution was used as mobile phase, the detection wavelength, column temperature, flow rate, and sample volume were 210 nm, 60°C, 1 mL/min, and 10 *μ*L, respectively.

### 3.2. Standard Curves

**Figure 1:**
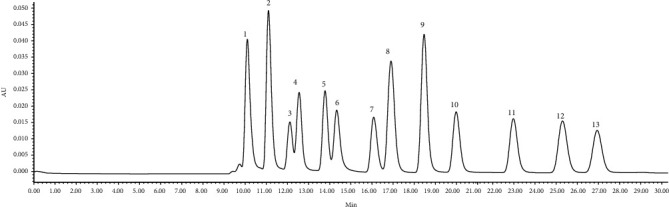
HPLC chromatography of mixed standards. Standards: oxalic acid (1), maleic acid (2), citric acid (3), tartaric acid (4), malic acid (5), quinic acid (6), succinic acid (7), fumaric acid (8), formic acid (9), acetic acid (10), propionic acid (11), isobutyric acid (12), and butyric acid (13).

At least seven concentrations of standard compounds were diluted and analyzed with three injections. The HPLC chromatography of the standard compounds is illustrated in Figure 1. Thirteen organic acids reached the baseline separation (The concentrations of each organic acid illustrated in Figure 1 were 120, 12.5, 410, 430, 900, 1000, 1000, 17.5, 625, 625, 625, 625, and 625 *μ*g/mL, respectively.). Standard curves of the investigated components were established by plotting the peak areas (Y) versus the concentration of each standard compound (X, *μ*g/mL). The correlation coefficient and test range are listed in Table 1.

### 3.3. Limits of Detection and Quantification

The limits of detection (LOD) under the chromatographic conditions were determined at the lowest detectable concentration with a signal-to-noise ratio (S/N) greater than three, and the limits of quantification (LOQ) was determined at the lowest concentration with an S/N greater than ten (Table 1).

### 3.4. Repeatability Experiment

The standard solution was sampled in five replicates and determined through chromatography. The results showed that the calculated RSD values of the peak area were within the range of 0.43%-2.13%, indicating good repeatability (Table 2).

### 3.5. Reproducibility Experiment

The mixed standard solutions were prepared parallelly within the concentration range of the standard curves for five times. The RSD values were within the range of 0.67%-2.22%, indicating stable reproducibility (Table 2).

### 3.6. Stability Experiment

The mixed standard solution was determined at 0, 2, 4, 10, 15, and 20 h, respectively. The RSD values of the retention time were within the range of 0.05%-0.30%, and the RSD values of the peak area were within the range of 0.74%-2.94%. This result suggested that the sample was stable within 20 h (Table 2).

### 3.7. Recovery Experiment

The recovery experiment was performed by adding 50%, 100%, and 150% of individual standards into a known concentration of mixed standards. One sample treated without standards was considered as the control. Then, these samples were prepared and analyzed through chromatography. The chromatography analysis was performed in triplicates at each level. The recovery rate was calculated by the standard amount detected and the standard amount added into the medium. The recovery rates of the investigated compounds were within the range of 91.9%-102%.

### 3.8. Analysis of Samples

**Figure 2:**
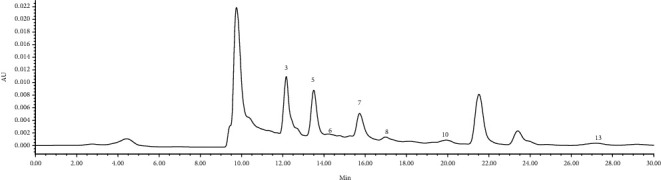
HPLC chromatograph of PDB culture medium. The compounds' numbers are the same as in Figure 1.

**Figure 3:**
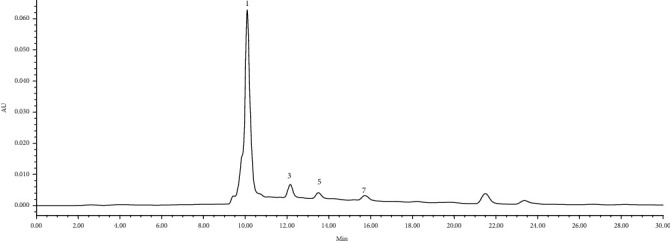
HPLC chromatograph in liquid medium of *L. edodes*. The compounds' numbers are the same as in Figure 1.

The contents of organic acids in the liquid medium of *L. edodes* and *F. velutipes* were analyzed. The results demonstrated that the sample solution of PDB (before incubation) contained citric acid, malic acid, quinic acid, succinic acid, fumaric acid, acetic acid, and butyric acid (Figure 2). There were two unidentified peaks with a *R_t_* of 21.5 min and 23.5 min, respectively, in addition to the expected samples. Oxalic acid, citric acid, malic acid, and succinic acid were detected in the sample solution of *L. edodes* (Figure 3 and Table 3). Organic acids in the liquid medium of *F. velutipes* are shown in Table 3. The contents of other organic acids were not detected. It is worth noting that the content of oxalic acid was hardly detected in PDB and increased after the growth of *L. edodes* or *F. velutipes*, indicating that oxalic acid was secreted during the growth of mycelia. The contents of oxalic acid, citric acid, malic acid, and succinic acid with large differences between the solutions of *L. edodes* and *F. velutipes* were detected. Similarly, only 4 types of organic acids were detected in the former, while 6 kinds were detected in the latter (Table 3). These results indicated that the types and contents of organic acids in the liquid medium of different edible fungi exhibit significant differences. It was also observed that citric acid, malic acid, and succinic acid contents showed a significant decrease after incubation of *L. edodes* mycelium. In contrast, these organic acids showed an increasing trend after incubation of *F. velutipes* mycelium in the same culture medium and culture environment. This might have happened due to the differences in the optimal pH required for the growth of different edible fungi. The edible fungus continuously secretes organic acids during the growth process to regulate the pH of its external environment, which could promote its own growth. In contrast, different edible fungi exhibit different abilities for decomposition and utilization of organic acids.

The standard column RP-C18 is mostly used to detect the organic acids, which is an alkyl chains bonded silica gel column. This can also be used to separate most of the organic compounds and has a wide range of applications. However, it shows a low efficiency in analyzing multiple organic acids. The current study results demonstrated that the sugar columns, such as SH1011 coupled with KC-811, were able to analyze around 13 organic acids simultaneously. Compared with the analytical columns commonly used in the current research [12, 24, 27, 28], the stationary phase of SH1011 and KC-811 used in this study exhibit a high-capacity cation exchange resin that is formed by the hydrogen-type sulfonated cross-linked polystyrene divinylbenzene copolymer, including Donnan repulsion, space repulsion, and adsorption processes. It also exhibits a good separation in analyzing organic acids. The systematic methodological investigation also suggests this method to be a better choice for analyzing multiple organic acids.

## 4. Conclusion

Our results concluded that the HPLC is the most convenient and reliable methodology for detecting organic acids due to excellent precision, stability, and capability of determining the organic acids in the liquid culture or the corresponding culture environment of the edible fungus for a long-term. Moreover, the utilization of the sugar columns, such as SH1011 coupled with KC-811, was capable of detecting 13 organic acids simultaneously, with a good separation. A guard column SH-G was used to remove protein from the sample and protect the columns from contamination. Compared to the existing research on the analysis of organic acids, the utilization of sugar columns in our methodology successfully analyzed around 13 organic acids simultaneously. In addition, the contents of organic acids in the culture medium of *L. edodes* and *F. velutipes* were also determined. Of which oxalic acid, citric acid, malic acid, and succinic acid were the major organic acids in the two samples. Therefore, this method could be widely used for the analysis of multiple organic acids and might provide a theoretical basis for exploring the mechanism behind the effects of organic acids on the growth of mycelium.

## Figures and Tables

**Table 1:** Linear regression data, LOD, and LOQ of the standard compounds.

Standard analytes	Linear regression data			LOD (*μ*g/mL)	LOQ (*μ*g/mL)
	Regressive equation	Test range (*μ*g/mL)	*R* ^2^		
Oxalic acid	*Y* = 5.67*e* + 003*X* − 9.61*e* + 003	4-1000	0.9997	1.71	6.53
Maleic acid	*Y* = 6.00*e* + 004*X* + 1.04*e* + 004	0.4-200	0.9991	0.05	0.10
Citric acid	*Y* = 5.99*e* + 002*X* − 5.25*e* + 003	10-2000	0.9998	3.23	12.93
Tartaric acid	*Y* = 9.89*e* + 002*X* − 2.47*e* + 003	10-2000	0.9999	3.36	7.73
Malic acid	*Y* = 4.80*e* + 002*X* − 4.96*e* + 003	10-2000	0.9999	3.53	14.13
Quinic acid	*Y* = 2.99*e* + 002*X* − 3.446*e* + 003	10-2000	0.9999	10.63	18.97
Succinic acid	*Y* = 3.37*e* + 002*X* − 2.82*e* + 003	10-2000	0.9999	8.31	15.82
Fumaric acid	*Y* = 4.60*e* + 004*X* − 5.53*e* + 003	0.4-200	0.9999	0.07	0.20
Formic acid	*Y* = 7.11*e* + 002*X* − 6.16*e* + 003	10-1000	0.9999	5.13	14.57
Acetic acid	*Y* = 3.51*e* + 002 *X* + 2.90*e* + 002	10-1000	0.9999	4.89	9.77
Propionic acid	*Y* = 3.46*e* + 002*X* − 2.00*e* + 003	10-1000	0.9999	4.89	12.06
Isobutyric acid	*Y* = 3.79*e* + 002*X* − 2.10*e* + 003	10-1000	0.9999	9.43	19.53
Butyric acid	*Y* = 3.23*e* + 002*X* − 9.69*e* + 002	10-1000	0.9999	4.75	9.63

**Table 2:** Data of precision, stability, reproducibility, and recovery.

Standard analytes	Repeatability RSD (%)	Stability		Reproducibility RSD (%)	Recovery (%)
		Peak area RSD (%)	Retention time RSD (%)		
Oxalic acid	0.43	1.19	0.16	1.65	102.0
Maleic acid	0.57	0.80	0.30	1.47	93.5
Citric acid	1.61	1.83	0.16	1.08	99.5
Tartaric acid	0.67	0.74	0.19	1.70	96.2
Malic acid	1.05	1.22	0.13	2.08	94.7
Quinic acid	1.15	1.75	0.11	1.23	93.8
Succinic acid	1.55	1.52	0.07	2.22	94.2
Fumaric acid	1.27	1.36	0.25	1.54	91.9
Formic acid	0.75	0.74	0.10	1.49	98.1
Acetic acid	2.13	1.77	0.05	1.12	101.8
Propionic acid	0.87	1.27	0.05	1.05	95.6
Isobutyric acid	1.19	1.16	0.05	1.38	94.4
Butyric acid	1.79	2.94	0.05	0.67	95.8

**Table 3:** Contents (*μ*g/mL) of analytes in culture medium of PDB and samples.

Analytes	PDB	*L. edodes*	*F. velutipes*
Oxalic acid	—^a^	145.21 ± 2.18^c^	283.52 ± 3.28
Citric acid	257.13 ± 3.22	160.92 ± 1.89	842.59 ± 4.46
Tartaric acid	+^b^	—	77.62 ± 2.17
Malic acid	289.18 ± 1.74	128.26 ± 1.10	825.18 ± 13.81
Quinic acid	47.40 ± 1.06	+	+
Succinic acid	290.51 ± 5.11	170.28 ± 3.84	736.18 ± 17.25
Fumaric acid	0.39 ± 0.01	—	3.67 ± 0.13
Acetic acid	47.85 ± 1.74	+	+
Butyric acid	43.28 ± 1.39	+	+

^a^Undetected. ^b^Under the limit of quantification. ^c^The data was presented as average of duplicates (*n* = 3).

## Data Availability

The data set supporting the conclusions of this study is available and we have agreed to share the data set. You can contact us by email to obtain the raw data in our manuscript.

## References

[1] Takao S. (1965). Organic acid production by Basidiomycetes. *Applied Microbiology*.

[2] Galkin S., Vares T., Kalsi M., Hatakka A. (1998). Production of organic acids by different white-rot fungi as detected using capillary zone electrophoresis. *Biotechnology Techniques*.

[3] Jarosz-Wilkolazka A., Graz M. (2006). Organic acids production by white rot Basidiomycetes in the presence of metallic oxides. *Canadian Journal of Microbiology*.

[4] Jarosz-Wilkolazka A., Gadd G. M. (2003). Oxalate production by wood-rotting fungi growing in toxic metal-amended medium. *Chemosphere*.

[5] Jones D. L., Dennis P. G., Owen A. G., van Hees P. A. W. (2003). Organic acid behavior in soils – misconceptions and knowledge gaps. *Plant and Soil*.

[6] Pohlman A. A., Mc Coll J. G. (1986). Kinetics of metal dissolution from forest soils by soluble organic acids. *Journal of Environmental Quality*.

[7] Rupa T. R., Tomar K. P., Srinivasa R. C., Subba R. A. (2001). Kinetics of phosphate sorption-desorption as influenced by soil pH and electrolytes. *Agrochimica*.

[8] Millet M., Wortham H., Sanusi A., Mirabel P. (1997). Low molecular weight organic acids in fogwater in an urban area: Strasbourg (France). *Science of the Total Environment*.

[9] Shimada M., Akamtsu Y., Tokimatsu T., Mii K., Hattori T. (1997). Possible biochemical roles of oxalic acid as a low molecular weight compound involved in brown-rot and white-rot wood decays. *Journal of Biotechnology*.

[10] Liu A. M., Chen J. F., Zhang J. J., Fang Q., Wang M., Zha Q. (2010). Effect of organic acid and amino avcid on the mycelial growth of *Agaricus bisporus* 2796. *Chinese Agricultural Science Bulletin*.

[11] Wang Y., Yu C. X., Cao H., Wang H., Chen M. J. (2015). Effect of oxalic, citric and tartaric acids on *Pleurotus eryngii* and *Flammulina velutipes* mycelial biomass production and DNA content. *Acta Edulis Fungi*.

[12] Ivanova-Petropulos V., Tašev K., Stefova M. (2016). HPLC method validation and application for organic acid analysis in wine after solid-phase extraction. *Macedonian Journal of Chemistry and Chemical Engineering*.

[13] Dinkci N., Akalin A. S., Gonc S., Unal G. (2007). Isocratic reverse-phase HPLC for determination of organic acids in Kargı Tulum cheese. *Chromatographia*.

[14] Peres R. G., Moraes E. P., Micke G. A., Tonin F. G., Tavares M. F. M., Rodriguez-Amaya D. B. (2009). Rapid method for the determination of organic acids in wine by capillary electrophoresis with indirect UV detection. *Food Control*.

[15] Castineira A., Pena R. M., Herrero C., Garcia-Martin S. (2002). Analysis of organic acids in wine by capillary electrophoresis with direct UV detection. *Journal of Food Composition and Analysis*.

[16] Castineira A., Pena R. M., Herrero C., Garcia-Martin S. (2000). Simultaneous determination of organic acids in wine samples by capillary electrophoresis and UV detection: optimization with five different background electrolytes. *Journal of High Resolution Chromatography*.

[17] Agius C., von Tucher S., Poppenberger B., Rozhon W. (2018). Quantification of sugars and organic acids in tomato fruits. *MethodsX*.

[18] Jham G. N., Fernandes S. A., Garcia C. F., Silva A. A. d. (2002). Comparison of GC and HPLC for the quantification of organic acids in coffee. *Phytochemical Analysis*.

[19] Belke C. J., Irwin A. J. (1992). Determination of organic acids in beer after extraction with an anion-exchange resin. *Journal of the American Society of Brewing Chemists*.

[20] Cao J., Hang Y., Lu J., Tong Z. (2010). Determination of nonnitrogenous organic acids and inorganic anions in sugarcane molasses and molasses alcohol waste by ion chromatography with solid-phase extraction. *Chinese Journal of Chromatography*.

[21] Ohira S. I., Kuhara K., Shigetomi A. (2014). On-line electrodialytic matrix isolation for chromatographic determination of organic acids in wine. *Journal of Chromatography A*.

[22] Geng X. M., Zhang S. F., Wang Q., Zhao Z. B. (2008). Determination of organic acids in the presence of inorganic anions by ion chromatography with suppressed conductivity detection. *Journal of Chromatography A*.

[23] Zaky A. S., Pensupa N., Andrade-Eiroa Á., Tucker G. A., Du C. (2017). A new HPLC method for simultaneously measuring chloride, sugars, organic acids and alcohols in food samples. *Journal of Food Composition and Analysis*.

[24] Pereira V., Camara J. S., Cacho J., Marques J. C. (2010). HPLC-DAD methodology for the quantification of organic acids, furans and polyphenols by direct injection of wine samples. *Journal of Separation Science*.

[25] He X. K., Xing Z. T., Rao Q. X., Zhao X. Y., Zhao M. W. (2012). Determination of organic acids in *Lintinus edodes* by HPLC. *Natural Product Research and Development*.

[26] Yang Y., Gu Z., Liu Y. F., Zhou S., Zhang J. S. (2013). Determination of seven organic acids in edible fungi by reversed-phase high performance liquid chromatography. *Mycosystema*.

[27] Bheemaiah M. M., Kushalappa B. A. (2019). Estimation and comparison of amount of organic acids from dried leaves of *Garcinia cambogia*, *Garcinia indica*, *Garcinia xanthochymus*, and *Garcinia morella* by high-performance liquid chromatography. *Pharmcognosy Research*.

[28] Fereshteh K., Hamideh A. (2014). Determination of ascorbic acid in different citrus fruits under reversed phase conditions with UPLC. *European Journal of Experimental Biology*.

[29] Raghav R., Yadav N., Tyagi G. (2012). Degradation studies of organic acids in commercially packed fruit juices: a reverse phase high performance liquid chromatographic approach. *International Journal of Food Engineering*.

